# Efficient Expression of Lactone Hydrolase Cr2zen for Scalable Zearalenone Degradation in *Pichia pastoris*

**DOI:** 10.3390/toxins18010010

**Published:** 2025-12-23

**Authors:** Mukhtar Ahmad, Hui Wang, Xiaomeng Liu, Shounan Wang, Tie Yin, Kun Deng, Caixia Lu, Xiaolin Zhang, Wei Jiang

**Affiliations:** 1 State Key Laboratory of Animal Biotech Breeding, College of Biological Sciences, China Agricultural University, Beijing 100193, China; mukhtar.micro@gmail.com (M.A.); wh15666529202@163.com (H.W.);; 2 COFCO Nutrition and Health Research Institute Co., Ltd., COFCO Corporation, Changping District, Beijing 102209, China; wsw_lxm2012@163.com (X.L.);; 3 College of Food Science and Pharmaceutical Engineering, Nanjing Normal University, Nanjing 210023, China

**Keywords:** zearalenone, degradation, hydrolase, *Pichia pastoris*, signal peptide, codon optimization, high-density fermentation

## Abstract

Zearalenone (ZEN) is a thermostable, lipophilic, non-steroidal estrogenic mycotoxin produced by *Fusarium* spp. that persistently contaminates food and feed. Its strong estrogenic activity and resistance to conventional detoxification strategies pose significant threats to food safety and human and animal health. Conventional physical and chemical degradation methods often compromise nutritional quality and leave toxic residues. Here we report the engineering of a novel *Clonostachys rosea* lactone hydrolase, Cr2zen, for efficient ZEN degradation in *Pichia pastoris* under mild conditions. Native Cr2zen exhibited a protein concentration of 0.076 mg/mL, achieving a degradation rate of approximately 17.9% within 30 min, with kinetic parameters of *K_m_* 75.9 µM and *V_max_* 0.482 µmol/L/s at 30 °C and pH 8.0. By integrating signal peptide screening and codon optimization, we identified Ser-Cr2 as the most effective variant, achieving a rapid 81.53% degradation of 10 ppm ZEN under mild conditions. Fed-batch cultivation in a 7.5 L bioreactor resulted in high cell densities of OD_600_ 332.8 for Ser-Cr2 and 310.8 for Oser-Cr2, with extracellular protein concentrations of 0.62 and 0.79 g/L, respectively. The results demonstrate that signal peptide engineering and codon optimization substantially improved the production of lactone hydrolase in *P. pastoris*. This study establishes a scalable ZEN degradation under mild conditions in *P. pastoris* and outlines a strategy to integrate protein and process engineering for enhanced enzymatic mycotoxin degradation.

## 1. Introduction

Mycotoxins are deleterious secondary metabolites synthesized by filamentous fungi under particular environmental conditions, notably elevated humidity and temperature [1]. The term “mycotoxin” originated from “mykes” signifying fungi, and “toxicon” denoting poison [2]. Globally, these toxins are linked to nearly one billion cases of illness and over 1.6 million deaths each year [3]. As of now, more than 400 chemically varied mycotoxins have been recognized, predominantly derived from fungal taxa like *Fusarium*, *Penicillium*, *Aspergillus*, *Streptomyces*, and *Alternaria* [4,5,6,7]. Among them, Zearalenone (ZEN) is one of the most significant *Fusarium*-derived mycotoxins [8], produced by *F. graminearum*, *F. oxysporum*, *F. equiseti*, *F. nivalis*, *F. crookwellense*, *F. sporotrichioides*, and *F. culmorum* [9,10,11,12]. Statistical investigations revealed that over 46% of worldwide food and feed are contaminated with ZEN, with peak amounts attaining 3049 µg/kg [13].

ZEN and its metabolites present significant toxicological hazards to humans and animals [14,15]. As a non-steroidal estrogen, it interacts with estrogen receptors and induces reproductive disorders in both genders [16,17,18,19]. Chronic exposure has been associated with hormone-dependent disorders, including cervical, ovarian, breast, and prostate cancers [20,21,22,23,24]. The International Agency for Research on Cancer (IARC) has categorized ZEN as a Group 3 carcinogen [25]. Furthermore, ZEN can be transformed into α-zearalenol in infected plants, a metabolite exhibiting about 90-fold increased estrogenic activity [26].

Due to its stability and global occurrence, the development of effective and safe ZEN degradation strategies remains a critical research priority [27]. Current methodologies are primarily classified into physical, chemical, and biological techniques [28]. Physical and chemical methods compromise food quality and induce secondary contamination, result in hazardous residues, and have limited efficacy [29,30,31]. Consequently, biological degradation is regarded as a highly promising advanced technique, particularly due to its specificity, environmental compatibility, and operational simplicity [29,32,33]. Several enzyme classes have been shown to degrade ZEN, including peroxidases, laccases, and lactonases [34]. Peroxidases and laccases operate via oxidative mechanisms and may produce incomplete degradation products, whereas lactonases directly hydrolyze the ZEN lactone ring, yielding non-toxic or less harmful metabolites [35]. Among these, ZHD101 from *Clonostachys rosea* IFO 7063 is one of the most extensively studied lactonases [36] and has been successfully expressed in *Escherichia coli* [37], *P. pastoris* [38], *Saccharomyces cerevisiae* [36], and *Lactobacillus reuteri* [39]. However, ZHD101 and its homologs, including ZHD518 [40], ZENG [41], ZEN-jjm [42], and ZHD_LD [43], still suffer from a limited pH range, low thermostability, and commercial utility. These limitations underscore the continued need for ZEN-degrading enzymes with higher catalytic efficiency, improved stability, and scalable production capabilities.

*P. pastoris* is an attractive host for heterologous expression of ZEN-degrading enzymes due to its strong *AOX1* promoter, capacity for high-density fermentation, efficient protein processing, and ability to secrete recombinant enzymes into the culture medium [13,44]. This system has been widely applied to produce lactonases and other industrial enzymes [35,45,46]. Expression efficiency can be further improved through codon optimization, the addition of secretion signal peptides, and the use of native propeptides [47,48]. Codon optimization enhances compatibility with host-preferred codons and can improve translation efficiency [49,50], while signal peptide engineering promotes extracellular secretion and higher protein yields [51,52]. Despite these advances, many ZEN-degrading enzymes exhibit inadequate stability or insufficient secretion efficiency in *P. pastoris*, limiting their industrial applications [53,54].

In response to these challenges, we identified a novel lactone hydrolase gene, *Cr2zen*, from the NCBI database, exhibiting 98.48% amino acid identity with Zhd101. To enhance its functional expression in *P. pastoris*, we synthesized the gene and employed two rational engineering strategies: (i) substitution of the native signal peptide with the human serum albumin signal to improve secretion, yielding the construct Ser-Cr2, and (ii) codon optimization for *P. pastoris* expression, resulting in Oser-Cr2. The constructs were heterologously expressed and characterized by SDS-PAGE, protein quantification, and HPLC-based ZEN degradation assays, demonstrating efficient activity under mild conditions (30 °C, pH 8.0). High-density fermentation further increased extracellular protein titers. This study integrates protein engineering with process scale-up in *P. pastoris* to establish a viable pathway for industrial ZEN degradation and to address key challenges related to secretion and enzyme stability.

## 2. Results and Discussion

### 2.1. Gene Cloning and Sequence Analysis

In this study, *P. pastoris* was selected for *Cr2zen* expression owing to its capacity for elevated expression levels, effective secretion, post-translational modifications, and appropriate protein folding [55]. Its ability to grow to high cell densities in simple, low-cost basal salt media [56] makes it a highly efficient and robust host for industrial-scale fermentation [57,58]. To augment the expression of the *Cr2zen* gene in *P. pastoris*, codon optimization was conducted to align more effectively with the codon use bias of *P. pastoris*, hence enhancing translational efficiency and maximizing protein expression levels. This improvement enhanced the Codon Adaptation Index (CAI) to 0.93 and reduced the GC content to 43.69%. Optimization also improved mRNA stability by removing inhibitory stem-loop structures that could hinder ribosome binding. Moreover, detrimental peaks were corrected to prolong the mRNA half-life, and adverse *cis*-acting regions were found and effectively altered to further enhance expression.

The *Zhd101* gene, derived from *C. rosea*, has notable discrepancies in codon use relative to the expression host. These disparities may reduce translational expression, a difficulty that can be mitigated through codon optimization. In the past, codon optimization has been effectively utilized in multiple instances to improve heterologous gene expression [26]. Moreover, Zhd101 demonstrates catalytic activity in hydrolyzing ZEN, converting it into a less estrogenic compound. To identify a lactone hydrolase with improved activity, a BLAST server (BLAST+v2.17.0; https://www.ncbi.nlm.nih.gov/BLAST, accessed on 26 August 2024) and alignment analysis were conducted on amino acid sequences associated with Zhd101.

Approximately 100 sequences were aligned, exhibiting sequence similarity between 56.87% and 100%. The sequences comprised proteins designated as ZEN lactone hydrolases, with the bulk categorized as hypothetical proteins. The putative protein Cr2zen (GenBank accession number ACW19936.1) from *C. rosea* exhibited 98.48% sequence similarity with ZHD101, suggesting its potential as a lactone hydrolase. As a result, the gene encoding *Cr2zen* was synthesized with codon optimization to facilitate subsequent cloning.

The *Cr2zen* gene encompasses 792 bp and encodes a protein of 264 amino acids with a theoretical molecular mass of 29 kDa. The N-terminal 18 amino acids (MKWVTFISLLFLFSSAYS) were predicted as a signal peptide sequence utilizing the SignalP 6.0 server (DTU Health Tech, Technical University of Denmark, Lyngby, Denmark; http://services.healthtech.dtu.dk/services/SignalP-6.0/, accessed on 26 August 2024). The ProtParam program on the ExPASY service (SIB Swiss Institute of Bioinformatics, Lausanne, Switzerland; https://web.expasy.org, accessed on 26 August 2024) projected an isoelectric point (pI) of 5.13 for Cr2zen. According to prior understanding of *Zhd101* degradation of ZEN, the procedure entails the cleavage of the lactone ring, followed by the decarboxylation of the resulting molecule [33].

### 2.2. Signal Peptide Optimization of Cr2zen in P. pastoris

The yield of heterologous protein expression is a crucial factor influencing the effectiveness and application of the target enzyme [59]. *P. pastoris* is widely recognized as a leading expression system for recombinant enzyme production. Furthermore, multiple factors, such as codon optimization, the integration of secretion signals, and the gene dosage of the target protein, have been demonstrated to markedly affect the expression levels of heterologous proteins in *P. pastoris*. This organism secretes a restricted array of endogenous proteins, thereby enhancing the purification of recombinant proteins, which can be effectively directed to the culture supernatant through signal peptide-mediated secretion [60]. The secretion of heterologous proteins in *P. pastoris* is fundamentally reliant on the presence of an N-terminal signal peptide [61], which enables the nascent protein’s entry into the endoplasmic reticulum for appropriate folding and secretion [62].

Bioinformatic analysis of the *Cr2zen* coding sequence utilizing SignalP 6.0 indicated the absence of a native eukaryotic signal peptide. This indicates that *Cr2zen* is devoid of inherent ER-targeting sequences, which probably accounts for its inadequate secretion in the *P. pastoris* system. To address this constraint, heterologous signal peptide replacement was utilized as a way to improve the extracellular secretion of *Cr2zen*. Three signal peptides were assessed for their ability to facilitate the secretion of recombinant proteins in *P. pastoris*: (i) the prepro-leader sequence of the *S. cerevisiae* α-mating factor [63], (ii) the signal peptide of *S. cerevisiae* invertase [64], and (iii) the signal peptide of human serum albumin [65]. These signal peptides have been documented to enhance the extracellular secretion and overcome processing limitations in yeast expression systems [66].

**Figure 1:**
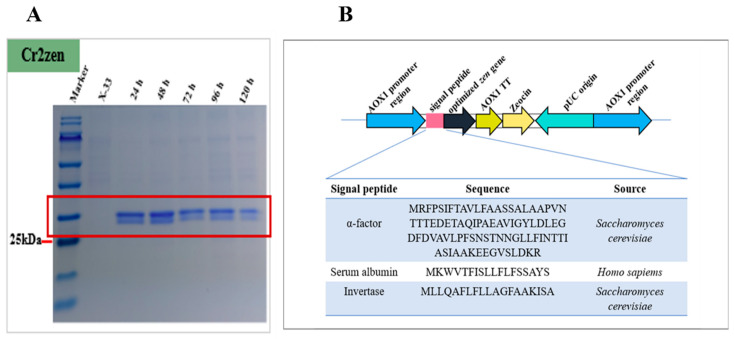
Expression of native Cr2zen and design of secretion signal variants in *P. pastoris*. (**A**) SDS-PAGE analysis of native Cr2zen expression in shake-flask cultures at different induction times. Lanes: molecular weight marker (10–180 kDa); X-33 as a control, and crude lysates. Observed bands are indicated with estimated molecular weights (kDa). Samples were collected at 24-h intervals (0–120 h). (**B**) Schematic depiction of the expression cassette under the *AOX1* promoter, showing insertion of different secretion signal peptides preceding the *Cr2zen* gene.

All three signal peptide sequences were codon-optimized based on the codon use bias of *P. pastoris* utilizing an in-house method to enhance translational efficiency [49]. The optimized sequences were then fused to the N-terminus of the *Cr2zen* open reading frame via overlap extension PCR employing appropriate primers (Table S1). Consequently, expression plasmids pPICZαA-*αCr2*, pPICZαA-*Ser-Cr2*, and pPICZαA-*Inv-Cr2* were regulated by the methanol-inducible *AOX1* promoter and afterwards transformed into *E. coli* DH5α for plasmid propagation and sequence validation. Subsequent to sequence verification, the plasmids were linearized using *Sac*I and transformed into *P. pastoris*. The integration into the AOX1 locus through homologous recombination was confirmed by colony PCR with AOX1 promoter and terminator-specific primers. The resultant recombinant strains α-Cr2, Ser-Cr2, and Inv-Cr2 (Figure 1B) were utilized for further expression analysis.

### 2.3. Enhanced Expression of Cr2zen in P. pastoris

**Figure 2:**
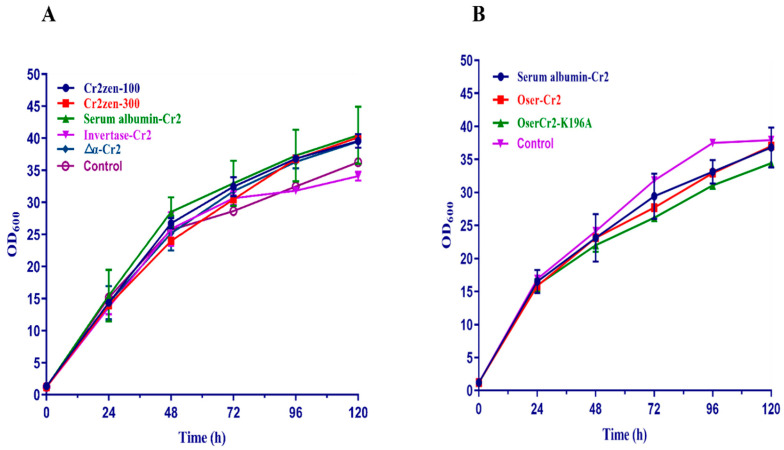
Growth curves of *P. pastoris* recombinant strains. (**A**) Growth curves of *P. pastoris* recombinant strains with signal peptide replacement during methanol induction. (**B**) Growth curves of *P. pastoris* recombinant strains expressing codon-optimized Oser-Cr2 and mutant variants during methanol induction.

Recombinant colonies were assessed on YPD agar plates containing Zeocin (100 µg/mL), and positive transformants were confirmed using PCR (Figure S1). Expression was regulated by the methanol-inducible P_AOX1_ promoter. Following 120 h of cultivation on BMMY medium, a notable secretion of the target protein was observed. The *P. pastoris* strains α-Cr2, Ser-Cr2, and Inv-Cr2 demonstrated extracellular protein concentrations of 0.086 ± 0.045 mg/mL, 0.036 ± 0.011 mg/mL, and 0.015 ± 0.001 mg/mL, respectively (Table S2). In contrast, shake flask culture of *S. cerevisiae* has been reported to contain 0.008 mg/mL of recombinant protein, like the modified AaeUPO [67]. This evidence suggests that our signal peptide variants supported significantly greater secretion levels. Given that distinct peptides can have a substantial impact on the efficacy of protein secretion in yeast expression systems. Throughout the induction phase, all recombinant strains exhibited sustained cellular proliferation without any discernible growth inhibition or cytotoxicity compared to the control strain, suggesting that the substitution of the native α-factor signal peptide did not negatively impact host physiology (Figure 2A).

**Figure 3:**
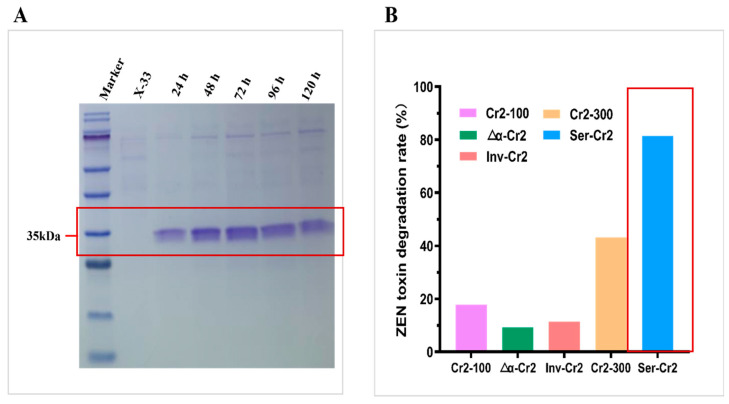
Expression and activity of engineered Ser-Cr2 in *P. pastoris*. (**A**) SDS-PAGE analysis of Ser-Cr2. Lanes: molecular weight marker (10–180 kDa), X-33 as a control, and crude lysates. Observed bands are indicated with estimated molecular weights (kDa). Samples were collected at 24-h intervals (0–120 h). (**B**) ZEN degradation efficiency by signal peptide-engineered enzymes in shake flask cultures.

Despite α-Cr2 exhibiting substantial extracellular protein concentration through quantitative assessment, SDS-PAGE evaluation indicated that Ser-Cr2 drove the most prominent ~35 kDa band (Figure 3A), consistent with the expected molecular mass. Conversely, the bands from α-Cr2 and Inv-Cr2 were not as prominent or were undefined (Figure S2). This contrast indicates that, whereas α-Cr2 secreted a greater total amount of protein, a larger proportion may have been misfolded, aggregated, or tainted with other host proteins, whereas Ser-Cr2 released a higher fraction of well-folded and stable enzyme. The protein content of the transformant cultures attained 0.076 mg/mL after 72 h of methanol induction in shaken flasks, consistent with documented yields for hydrolases derived from a yeast system [68].

The functional activity of the recombinant enzymes was evaluated using HPLC to quantify ZEN degradation, with a substrate concentration of 70.59 ± 2.74 ng/mL (Table S3). Under ideal reaction conditions (30 °C, pH 8.0), the enzyme from the serum albumin signal peptide strain, Ser-Cr2, exhibited superior catalytic performance, breaking down 81.53% of 10 ppm ZEN after 30 min. This represents an almost five-fold enhancement compared to the native Cr2zen-100 strain, which attained about 17.9% degradation, lowering ZEN concentrations to 313.68 ± 5.19 ng/mL. The Cr2zen-300 variant exhibited moderate activity, reducing ZEN concentration to 216.97 ± 0.80 ng/mL, signifying 43.2% degradation, whereas the invertase signal peptide-fused strain (Inv-Cr2) and the Δα-factor construct attained reductions of 11.5% (338.29 ± 5.11 ng/mL) and 9.4% (346.46 ± 4.94 ng/mL), respectively, suggesting relatively inferior enzymatic efficiency (Figure 3B). This finding is significant since Ser-Cr2, despite its reduced secretion output compared to α-Cr2, exhibited enhanced substrate turnover. This indicates that the selection of signal peptides can affect not only extracellular expression levels but also the enzyme’s folding, stability, and catalytic efficacy [69]. Similar impacts of leader sequences on enzymatic activity have been documented for additional heterologous proteins produced in *P. pastoris* [60,61,70]. The engineering of ZEN-degrading enzymes to improve catalytic performance is of considerable attention for agro-biotechnological applications [36].

The Cr2zen enzyme is a member of the lactone hydrolase family, known to catalyze the hydrolytic cleavage of the lactone ring in ZEN molecules, therefore reducing or abolishing their estrogenic effects. The enzymatic activity demonstrated against ZEN suggests that Cr2zen likely operates via a mechanism akin to previously identified ZEN lactonases. The catalytic mechanism of these enzymes often entails a nucleophilic attack on the carbonyl carbon of the lactone ring, leading to ring cleavage and the formation of a non-toxic linear hydrolysis product. We propose that Cr2zen may degrade ZEN in a similar manner consistent with studies reporting the breakdown of ZEN by other lactone hydrolases [46,71,72,73].

*P. pastoris* can utilize methanol as its sole carbon and energy source, with the initial and rate-limiting step of the methanol catabolic pathway mediated by an enzyme regulated by the P*_AOX1_* promoter. This promoter is regulated stringently by coordinated *cis*-regulatory sequences and *trans*-acting factors [74]. Methanol significantly enhances P_AOX1_ expression, while glucose suppresses it; transitioning the carbon supply from glucose to methanol initially derepresses and subsequently activates the promoter [75]. The elevated expression of Cr2zen in *P. pastoris* under the P_AOX1_ promoter highlights its efficacy for recombinant protein production and mitigates metabolic burden during the biomass growth phase [76]. The α-Cr2 attained the maximum extracellular yield, indicating that the selection of signal peptide markedly affected secretion efficiency, although the often-utilized α-mating factor signal from *S. cerevisiae* frequently results in enhanced secretion performance in *P. pastoris* [52,70]. Targeted mutations have been demonstrated to modulate secretion by alleviating mis-sorting and diminishing the unfolded protein response, hence enhancing productive secretion [60]. *P. pastoris* is an appropriate host, and the selection of signal peptides underscores the significance of improving secretion efficiency.

### 2.4. Enzymatic Characterization of Lactone Hydrolase Cr2zen

ZEN lactone hydrolases are predominantly neutral enzymes with minimal adaptability to pH fluctuations, particularly in acidic conditions. These enzymes typically exhibit peak catalytic activity at temperatures ranging from 30 °C to 40 °C and in alkaline conditions, with an optimal pH range of 8.0 to 10.0 [34]. Understanding the functional characteristics of the recombinant lactonase Cr2zen is essential for evaluating its applicability in biocatalytic processes for the effective breakdown of the estrogenic mycotoxin ZEN, particularly under settings pertinent to industrial biotechnology. The pure Cr2zen demonstrated peak enzymatic activity at pH 8.0 when utilizing ZEN as a substrate.

**Figure 4:**
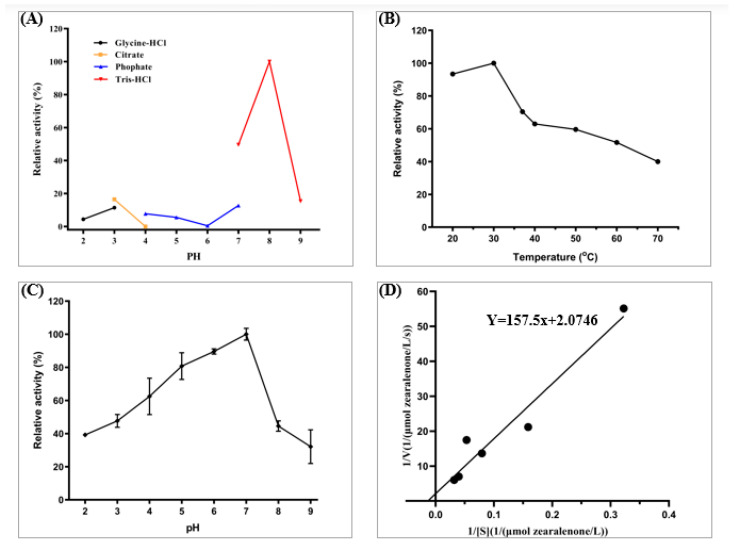
Biochemical characterization of native Cr2zen expressed in *P. pastoris*. (**A**) Effect of different buffer systems (Glycine-HCl, Citrate, Phosphate, and Tris-HCl) on Cr2zen activity at varying pH values. (**B**) Temperature profile of Cr2zen activity (20–70 °C). (**C**) Determination of the pH stability, with stability observed around pH 7.0. (**D**) kinetic parameters of Cr2zen toward ZEN.

The lactonase Cr2zen exhibited pH-dependent activity, achieving maximal catalytic efficiency (100% relative activity) in Tris-HCl buffer at pH 8.0 (Figure 4A). This behavior reflects a classical bell-shaped pH-activity profile typical of hydrolases, with activity sharply declining to below 20% in acidic Glycine-HCl (pH 2.0–3.0) and Citrate (pH 4.0–5.0) buffers, likely due to the protonation of catalytic residues. Furthermore, alkaline inactivation above pH 8.5 indicated potential conformational changes or deprotonation of the nucleophile. This optimum pH corresponds with the pH preference noted for CbZHD from *C. bantiana* [36], as well as for ZHD518 from *R. mackenziei* expressed in *E. coli* [34], and ZHD607 from *P. americana* expressed in *P. pastoris* [71].

**Figure 5:**
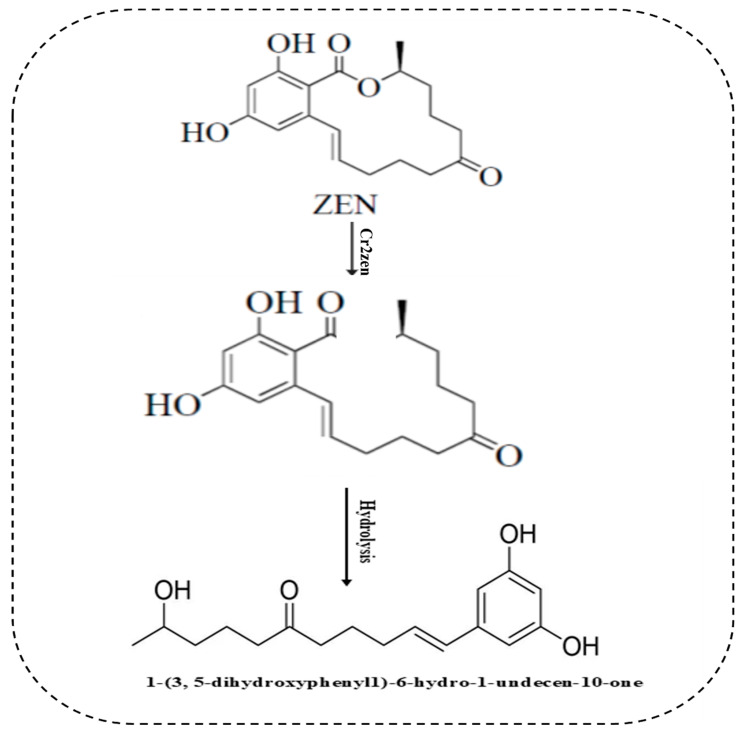
Proposed mechanism of ZEN degradation by the Cr2zen lactone hydrolase. The enzyme is hypothesized to catalyze the hydrolytic cleavage of the ZEN lactone ring, leading to the formation of a non-toxic open-ring product. The schematic is based on previously reported mechanisms of ZEN lactonases and illustrates a possible degradation pathway for Cr2zen.

The pH stability assays demonstrated remarkable structural integrity in Tris-HCl pH 7.0 (Figure 4C), exhibiting over 90% residual activity after 1 h incubation. In contrast, extreme pH conditions (≤5.0 or ≥9.0) induced irreversible denaturation, resulting in less than 40% activity. Temperature profiling determined 30 °C as the optimal temperature (Figure 4B), with activity significantly declining above 40 °C (maintaining only 50% activity at 50 °C), indicating a mesophilic characteristic corroborated by Arrhenius plot discontinuities that suggest thermal denaturation. The enzyme’s consistent activity within a moderate pH and temperature range reflects the conditions commonly seen in industrial fermentation and degradation processes. Kinetic analysis through Lineweaver–Burk transformation (R^2^ = 0.99) produced a *K_m_* of 75.9 ± 2.1 µM and a *V_max_* of 0.482 ± 0.015 µmol/L/s (Figure 4D), indicating moderate substrate affinity. The elevated *K_cat_*/*K_m_* ratio (6.35 × 10^3^ M/s) reflects moderate substrate affinity suitable for ZEN concentrations commonly encountered in experimental and applied degradation settings (Figure 5).

Recombinant Cr2zen maintained high activity at neutral pH and moderate temperatures, exhibiting maximum activity at pH 8.0 and 30 °C. Extremely high temperatures (>40 °C) or acidic environments (≤5.0) significantly decreased activity. Other ZEN-degrading lactonases, in contrast, usually exhibited their best activity between 37–45 °C and pH 8.0–10.0 [13], suggesting that Cr2zen provides better stability in neutral conditions that are appropriate for industrial processing. While lactonase like ZHD-P maintained an 80% activity across pH 7.0–9.0 [77], Cr2zen demonstrated exceptional pH stability, maintaining over 90% activity after 1 h at pH 7.0, suggesting that, at neutral pH, Cr2zen is quite stable. Compared to ZenH *K_m_* and *V_max_* 12.64 ± 0.16 µM and 0.2 ± 0.012, respectively [24], Cr2zen displayed a higher *K_m_*, indicating a lower substrate affinity, while the lower *V_max_* of ZenH than Cr2zen indicates a higher catalytic turnover capability of Cr2zen.

These biochemical findings establish a foundational basis for the incorporation of Cr2zen into enzyme-mediated degradation. The enzymatic characterization of Cr2zen underscores its potential as a viable candidate for the enzymatic mitigation of ZEN, paving the way for its incorporation into engineered microbial systems and transgenic platforms to improve biocatalytic efficacy in various biodegradation applications.

### 2.5. High-Density Fermentation of Strain Ser-Cr2

**Figure 6:**
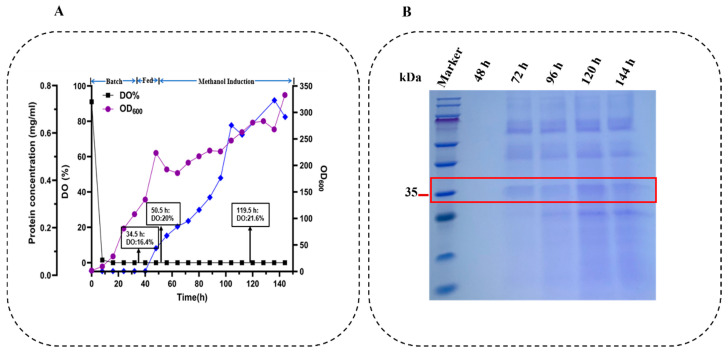
High-density fermentation and expression analysis of Ser-Cr2 in P. pastoris. (**A**) Fed-batch fermentation profile showing cell growth (OD_600_), dissolved oxygen (DO%), and blue line indicate protein concentration during methanol induction. (**B**) SDS-PAGE analysis of fermentation supernatants collected at different induction times (48–144 h). Lanes: molecular weight marker (10–180 kDa) and crude lysates. Observed bands are indicated with estimated molecular weights (kDa).

Due to the exceptional catalytic efficacy of Ser-Cr2 demonstrated in shake flask fermentation, this strain was chosen for scale-up in a 7.5 L high-density fermentation system. The OD_600_ reached 332.8 after 144 h (Figure 6A). This significant increase compared with shake flask culture primarily results from superior bioreactor control, including efficient oxygen transfer, precise feed-rate adjustment, and stable pH control [78]. The stable DO pattern observed during fermentation coincided with cell growth and enhanced secretion of the recombinant protein [79]. Moreover, appropriate regulation of carbon source availability minimizes substrate inhibition and prevents ethanol accumulation, thereby supporting cell growth [80]. The substantial culture volume and mechanical mixing facilitate homogeneous nutrient distribution, promoting uniform cell development to elevated biomass densities [78].

The extracellular Ser-Cr2 protein concentration attained 0.62 g/L, signifying a significant enhancement compared to shake flask conditions, attributable to augmented cell biomass facilitating greater total protein expression capacity, along with more uniform methanol induction in the regulated fermenter environment. Recombinant protein yield increases with biomass accumulation; however, this relationship holds only when induction does not trigger metabolic stress. Stress conditions can activate the unfolded protein response or enhance proteolysis in *P. pastoris*, ultimately reducing secretion efficiency [81]. The induction phase of fermentation is enhanced by stable pH, dissolved oxygen, and methanol concentrations, facilitating effective transcription from the P*_AOX1_* promoter and continuous secretion of the target protein [82]. Furthermore, diminished shear stress and precise temperature regulation in the fermenter mitigate stress-induced misfolding, thereby enhancing the ratio of properly folded, active enzyme in the culture supernatant [83].

SDS-PAGE analysis demonstrated a diverse protein profile, characterized by several non-target bands, suggesting the co-secretion of host proteins (Figure 6B). Functional HPLC evaluation indicated that the crude enzyme degraded merely 15.27% of 10 ppm ZEN within 30 min, significantly below the performance shown in shake flask experiments. This mismatch indicates that, despite elevated biomass and protein content, some Ser-Cr2 was broken down or misfolded during fermentation. In high-density *P. pastoris* cultures, extended fermentation may result in the proteolytic cleavage of recombinant proteins due to the overexpression and release of endogenous proteases from lysed cells [84]. Similar observations were reported in a recombinant protein production process, where prolonged high-density fermentation significantly increased proteolytic degradation [85].

Furthermore, protein misfolding under prolonged induction stress may lead to aggregation or incorrect disulfide bond formation, reducing enzymatic activity despite high expression yields [86]. Additionally, methanol metabolism results in formaldehyde and hydrogen peroxide, which may induce oxidative damage to vulnerable proteins during prolonged induction [87]. These factors, whether individually or synergistically, presumably contributed to the diminished functional activity of Ser-Cr2 in the high-density fermenter, despite the increased total protein production.

### 2.6. Codon-Optimized Oser-Cr2 for Enhanced Expression

Analysis of the Ser-Cr2 sequence identified several infrequent codons with low usage frequency in *P. pastoris*, which are recognized to hinder translational efficiency and impair proper folding due to limited cognate tRNA [88]. The complete open reading frame was meticulously re-engineered to align with *P. pastoris* codon usage bias, yielding the fully codon-optimized variant Oser-Cr2 (Figure S3). The optimization modified GC content to align with the optimal range for yeast production and eliminated putative inhibitory patterns, including cryptic splice sites and AT-rich sequences [89].

Bioinformatic analysis of the codon-optimized Oser-Cr2 identified a conventional Kex2 protease recognition motif within the polypeptide sequence (Figure S4). In *P. pastoris*, Kex2 is essential for processing secretory proteins by cleaving after lysine or arginine residues. This processing can promote proper protein maturation; however, improper cleavage within the mature protein region may lead to partial degradation, diminished activity, or decreased secretion yields [90]. We predicted that the K-196 location could be vulnerable to Kex2-mediated cleavage, leading to the instability of Oser-Cr2 in *P. pastoris*.

To test this hypothesis, site-directed mutagenesis was performed to replace Lys-196 residue with alanine, thereby eliminating the predicted Kex2 recognition site. Specific primers were designed, and the recombinant plasmid was transformed into *E. coli* DH5α, subsequently inserted into *P. pastoris* following sequencing, and confirmed using colony PCR (Figure S5). The recombinant *P. pastoris* strains expressing the codon-optimized Oser-Cr2 and its K196A mutant variant were cultured under methanol-inducible conditions to evaluate their protein expression. All strains exhibited normal growth kinetics throughout the induction phase, demonstrating that neither codon optimization nor site-directed mutagenesis introduced any observable metabolic burden or growth inhibition (Figure 2B). Quantitative protein analysis indicated that the Oser-Cr2 strain attained the maximum extracellular protein concentration of 0.125 mg/mL, signifying a notable enhancement compared to Ser-Cr2, while the K196A mutant exhibited a markedly decreased yield (Table S4). This indicates that, although the mutation effectively eliminated the Kex2 site, it may have affected protein folding, secretion efficiency, or stability in the extracellular milieu [91].

**Figure 7:**
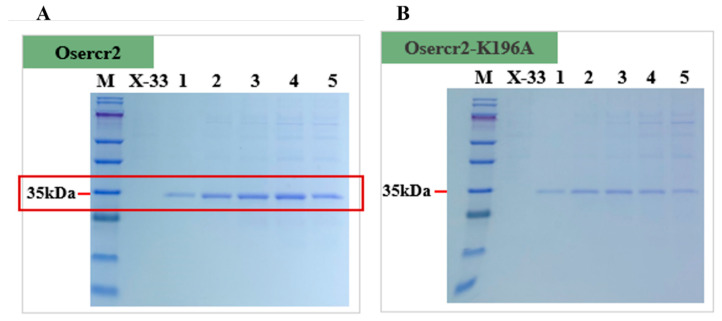
SDS-PAGE analysis of recombinant *P. pastoris* strains. (**A**) SDS-PAGE analysis of the Osercr2 strain showing clear protein bands corresponding to the target enzyme. (**B**) SDS-PAGE analysis of the mutant strain Osercr2-K196A with kex-2 restriction site mutation with shallower protein bands. Lanes: M; marker (10–180 kDa); X-33 as ontrol; 1; 2; 3; 4; 5, crude lysates. Observed bands are indicated with estimated molecular weights (kDa). Samples were collected at 24-h intervals (0–120 h).

The SDS-PAGE analysis of the induced culture supernatants confirmed these findings. The Oser-Cr2 had a distinct, well-defined band at the anticipated molecular mass (Figure 7A), reflecting higher expression levels, while the K196A mutant band was noticeably weaker (Figure 7B). The diminished intensity corresponds with the reduced protein concentration measurement, indicating that the elimination of the Kex2 site, despite possibly inhibiting proteolytic cleavage, may have caused structural modifications or compromised secretion efficiency in *P. pastoris*.

The findings suggest that the Kex2 cleavage motif at Lys-196 is not a principal factor in the degradation of Oser-Cr2 in *P. pastoris* under the tested conditions. The reported reduction in expression due to mutation suggests that the motif may have an indirect role in secretion or folding, maybe through contact with the endogenous processing machinery [92]. These results drive subsequent protein engineering techniques focused on achieving a balance between protease resistance and optimal secretion efficiency in *P. pastoris*.

### 2.7. High-Density Fermentation of Oser-Cr2 in Pichia pastoris

Based on the exceptional expression performance noted in shake flask cultivation, the codon-optimized Oser-Cr2 strain was chosen for scale-up in a 7.5 L high-density fermentation to enhance biomass yield and recombinant protein production. Fermentation was carried out in a controlled bioreactor setting, with dissolved oxygen meticulously managed through dynamic regulation of aeration rate and agitation speed to avoid oxygen constraint during the exponential growth phase.

**Figure 8:**
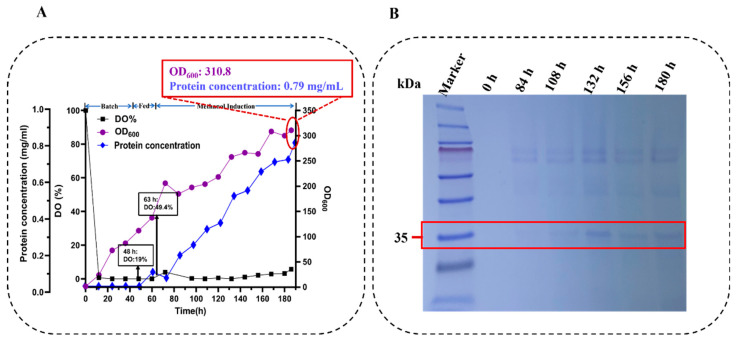
High-density fermentation and protein expression analysis of Oser-Cr2 in *P. pastoris*. (**A**) High-density fermentation profile showing dissolved oxygen (DO%), OD_600_, and protein concentration over time, reaching a final OD_600_ ~310 and protein concentration of ~0.79 mg/L. (**B**) SDS-PAGE analysis of Oser-Cr2 expression at different time intervals during high-density fermentation. Lanes: molecular weight marker (10–180 kDa) and crude lysates. Observed bands are indicated with estimated molecular weights (kDa).

Induction commenced after 63 h, coinciding with a noted increase in DO after the depletion of the primary carbon substrate, aligning with the transition to methanol-based metabolism in *P. pastoris* [87]. During the induction phase, the inducer-substrate feed was adjusted to sustain a stable concentration within the optimum range for P*_AOX1_* promoter activation while reducing substrate inhibition and formaldehyde buildup [93]. Following 186 h of growing, the culture attained an OD_600_ of 310.8, with an extracellular protein content of 0.79 g/L, indicating a significant enhancement compared to the shake flask yield (Figure 8A).

The significant increase in recombinant protein titer can be ascribed to the synergistic effects of elevated cell density, regulated induction parameters, and uniform nutrition distribution in the stirred-tank reactor setting [83]. Nonetheless, despite these advancements, Tricine-SDS-PAGE evaluation of culture supernatants indicated the breakdown of the target protein during the later phases of fermentation (Figure 8B).

The noted reduction in intact protein content is probably due to multiple factors. As biomass reaches elevated cell densities, the efficiency of oxygen transfer may diminish, even with active dissolved oxygen regulation [80], resulting in temporary hypoxic zones that induce metabolic stress on the cells [94]. In addition, late-stage cultures are susceptible to autolysis caused by mechanical shear, nutritional depletion, and the buildup of toxic byproducts, leading to the release of vacuolar and cytoplasmic proteases into the extracellular environment [95]. These proteases, upon secretion or leakage from disrupted cells, might catalyze the partial cleavage of heterologous proteins, thereby reducing the proportion of functionally active enzyme [96].

Furthermore, prolonged methanol induction produces reactive oxygen species, including hydrogen peroxide, which may cause oxidative changes or instability of vulnerable protein domains [97]. In the particular case of Oser-Cr2, these stresses may collaboratively lead to structural instability, causing partial proteolysis and misfolding. This behavior aligns with prior reports indicating that while high-density fermentation optimizes total protein yield, the functional quality of the produced protein may diminish if induction stress is not meticulously regulated [78].

Collectively, our findings indicate that, although the Oser-Cr2 strain attains enhanced expression levels during high-density fermentation, process-related stressors in the late induction phase present considerable obstacles in maintaining enzyme integrity. Future optimization should incorporate approaches such as protease-deficient host strains, the addition of protease inhibitors, refined methanol feeding profiles, and improved oxygen transport to reduce post-secretion destruction. Such methodologies may be essential for fully harnessing the enzymatic capabilities of Oser-Cr2 for industrial-scale ZEN degradation.

## 3. Conclusions

Zearalenone is a persistent estrogenic mycotoxin that presents significant threats to agriculture, environmental health, and food safety. We engineered the *C. rosea* lactone hydrolase Cr2zen in *P. pastoris* to facilitate scalable degradation of ZEN under mild conditions. The native Cr2zen exhibited a protein concentration of 0.076 mg/mL along with a favorable catalytic nature. Signal peptide replacement resulted in Ser-Cr2, which in shake-flask experiments attained 0.036 mg/mL and degraded 81.53% of 10 ppm ZEN within 30 min. High-density fermentation elevated protein production to 0.62 g/L at OD_600_ 332.8, but degradation decreased to 15.27% within 30 min. Codon optimization yielded Oser-Cr2, which rose from 0.125 mg/mL in shake flask cultures to 0.79 g/L in fed-batch settings at OD_600_ 310.8. These findings reveal a modifiable trade-off: Ser-Cr2 optimized catalytic efficiency, while Oser-Cr2 optimized yield. Bioreactor analyses further emphasize proteolysis, induction stress, and oxidative stress as critical limitations to functional stability.

Subsequent research should incorporate LC–MS/MS-based identification of ZEN degradation products, utilize protease-deficient hosts, enhanced induction methodologies, and sequence stability to maintain activity at scale. Validation within agricultural matrices and against essential ZEN metabolites will be crucial for translation. This study identifies a new lactone hydrolase variant and demonstrates its efficient secretory expression and scalable fermentation performance in *P. pastoris* under mild conditions, establishing an effective and scalable strategy for enzyme-based degradation. Although further optimization is needed for industrial application, these findings validate *P. pastoris* as a robust host for industrial enzyme production and provide a solid foundation for developing practical enzyme-based strategies for ZEN mitigation in food and feed systems.

## 4. Materials and Methods

### 4.1. Plasmids, Strains, Chemicals and Media

*E. coli* DH5α and *P. pastoris* X33 were utilized as hosts for gene cloning and heterologous expression, respectively, and were sourced from our laboratory. Plasmid extraction was conducted utilizing kits obtained from Tiangen Biotech (Beijing, China). PCR reagents and restriction endonucleases were procured from New England Biolabs (Ipswich, MA, USA). DNA markers were acquired from Real-Time Biotechnology Co. (Beijing, China). Protein markers were acquired from SMOBIO Technology, Inc. (Hsinchu, Taiwan). DNA polymerase, recombinase, and several associated enzymes were obtained from Vazyme Biotech Co. (Nanjing, China). The DNA primers were designed, and sequencing was conducted by Tsingke Biotechnology Co., Ltd. (Beijing, China). ZEN (purity > 99%), sourced from Sigma-Aldrich (St. Louis, MO, USA), was dissolved in dimethyl sulfoxide (DMSO) to yield a standard stock solution. Acetonitrile, employed as the organic phase in high-performance liquid chromatography (HPLC), was sourced from ANPEL (Shanghai, China). Methanol was of chromatographic grade purity, while water was purified with a Milli-Q academic water system (Millipore, Burlington, MA, USA). All supplementary chemicals and reagents utilized in this work were of analytical grade and were from commercial vendors. BMGY, BMMY, and YPD media were formulated in accordance with the guidelines specified in the *Pichia* Expression Kit (Invitrogen, Carlsbad, CA, USA). *E. coli* strains were cultivated and selected in diluted Luria–Bertani (LB) medium (Sigma-Aldrich, St. Louis, MO, USA).

### 4.2. Gene Cloning and Expression

The *Cr2zen* gene was codon-optimized to conform to the codon usage preferences of yeast utilizing the Java Codon Adaptation Tool (https://www.jcat.de, accessed on 26 August 2024). It was synthesized based on a hypothetical protein from *C. rosea* (GenBank accession number ACW19936.1) and subsequently cloned into the pPICZαA vector using *EcoR*I and *Not*I restriction sites, yielding the expression vector pPICZαA-Cr2zen (Figure 1B). The optimized gene was inserted into the pPICZαA vector, which includes the methanol-inducible AOX1 promoter and the α-factor secretion signal. The recombinant plasmid pPICZαA-Cr2zen was linearized with *Sac*I to facilitate the expression of the heterologous protein and subsequently introduced into *P. pastoris* X33 through electroporation, as previously documented [98]. Colonies were analyzed by PCR and confirmed through sequencing. The transformants were cultivated and screened on YPD solid plates supplemented with 100 µg/mL of Zeocin (Invitrogen, Corp; Carlsbad, CA, USA) and incubated for 48 h at 30 °C.

### 4.3. Enzyme Induction and Protein Analysis

The transformant with the highest activity was chosen and cultivated in 30 mL of YPD liquid media within a 100 mL conical flask at 30 °C for 48 h. Cells were collected from the fermentation broth via centrifugation (4000× *g*, 5 min), suspended in BMGY medium (5 mL tubes) for 48 h (30 °C, 180 rpm) until the optical density at 600 nm reached 4.0–6.0, and then resuspended in 200 mL of BMMY medium, containing 1.34% yeast nitrogen base (YNB) and 4.0 µg/mL biotin, and incubated at 30 °C for 96 h, with 1% methanol supplied every 24 h to stimulate protein expression. A 100 µL aliquot of the cell culture sample was obtained every 24 h, and protein expression was assessed utilizing 12% sodium dodecyl sulfate-polyacrylamide gel electrophoresis (SDS-PAGE) (Figure 1A). The protein concentration was measured via the Bradford assay, employing bovine serum albumin as the standard Bio-Rad protein assay kit (Bio-Rad Laboratories, Inc., Hercules, CA, USA) [99].

### 4.4. Optimization of Cr2zen Production

Due to the absence of an intrinsic signal peptide necessary for effective secretion of lactone hydrolase Cr2zen in *P. pastoris*, an approach for signal peptide replacement was implemented. The signal peptide sequence of lactone hydrolase was anticipated using the SignalP 6.0 server (DTU Health Tech, Technical University of Denmark, Lyngby, Denmark; http://services.healthtech.dtu.dk/services/SignalP-6.0/). Signal peptides from human serum albumin and *S. cerevisiae* invertase were chosen and modified according to the codon preferences of *P. pastoris*. Fusion PCR was employed to substitute the native sequence with the selected signal peptides utilizing appropriate primers (Table S1). The constructed plasmids (α-Cr2, Ser-Cr2, Inv-Cr2) were subsequently introduced into *P. pastoris* X-33 to yield high-efficiency secretory strains. Transformants were selected and validated via colony PCR using AOX-F/AOX-R primers and SDS-PAGE analysis to confirm the effective integration and secretion of the recombinant protein. To enhance the expression of the Ser-Cr2 strain in *P. pastoris*, codon optimization was executed to align the gene sequence with the preferred codon usage of *P. pastoris*. The optimization approach utilized the Codon Optimization Index (CAI) to exclude uncommon codons that may impede translation efficiency. The codon-optimized variant, named Oser-cr2, was synthesized and subsequently cloned into pPICZαA. The recombinant plasmid was later delivered into *P. pastoris* using electroporation, with transformants selected for protein expression triggered by methanol.

### 4.5. Expression of Cr2zen with High-Density Fermentation

The high-cell-density fermentation of *P. pastoris* was performed in strict accordance with the *Pichia* fermentation procedure (Invitrogen). The enhanced lactone hydrolase Cr2zen was produced in a 7.5 L fermentor (Shanghai Boxing Bio-engineering Equipment Co. Ltd., Shanghai, China), comprising 5 L of Basal Salts Medium (BSM) augmented with PTM1 solution as the primary fermentation medium. The fermentor conditions were maintained at a temperature of 30 °C and a pH of 5.5 using sodium hydroxide (50% *v*/*v*). The dissolved oxygen (DO) was regulated via aeration and agitation control, facilitating sustained exponential growth without oxygen limitation. The genetically engineered strain was initially cultivated using glycerol as the carbon source until the glycerol was entirely depleted from the medium. Dissolved oxygen levels were sustained at a low concentration by modulating the aeration rate between 4–13 L/min and altering the agitation speed from 400 to 700 rpm. An additional 200 mL of glycerol was administered during the subsequent feeding time. Following the increase in DO levels, a pure methanol solution containing PTM1 was introduced to enhance the synthesis of the target enzyme. Culture supernatant samples were obtained at 8 h intervals to assess cell density, protein content, and enzyme activity.

### 4.6. Zearalenone Degradation Assay

The optimum temperature range for lactone hydrolase activity on ZEN is documented as 37 °C to 45 °C, with the optimal pH recorded between 9 and 10 [37]. ZEN exhibited stability at pH 7.0; hence, the quantitative assessment of Cr2zen activity was performed at 30 °C and pH 8.0. To evaluate the degradation of ZEN by the Cr2zen enzyme, a standard reaction mixture was established, comprising 100 µM ZEN, 20 mM Tris-HCl buffer (pH 8.0), and a suitable quantity of the purified Cr2zen enzyme, totaling 1 mL in volume. The reaction was incubated at 30 °C with mild agitation. At specified time intervals (0, 1, 2, 4, 6, 8, and 24 h), 100 µL aliquots were collected, and the reaction was terminated by the addition of 10 µL of 1 M HCl. A linear association between ZEN concentration and peak area was established, facilitating the creation of a standard curve. One unit of ZHD activity is defined as the amount of enzyme necessary to facilitate the breakdown of 1 µg of ZEN per min under specified test conditions (pH 8.0, 30 °C).

### 4.7. HPLC Analysis

The concentration of ZEN in the reaction mixtures was measured using HPLC equipped with a fluorescence detector using a reverse-phase C18 column (Eclipse XDB-C18, 150 mm × 4.6 mm, 5 µm, Agilent Technologies, Santa Clara, CA, USA). The column temperature was sustained at 35 °C, with excitation and emission wavelengths set at 320 nm and 460 nm, respectively. The mobile phase employed a gradient system, initiating with 100% water for 4 min, subsequently transitioning linearly to 100% acetonitrile over 25 min, and maintaining 100% acetonitrile for 6 min at a flow rate of 0.8 mL/min. The aqueous phase comprised 0.6% (*v*/*v*) trifluoroacetic acid (TFA) in water, while the organic phase consisted of TFA in acetonitrile 0.5% (*v*/*v*). Both phases were degassed by ultrasonication for 30 min prior to use. Samples were filtered via a 0.22 µm membrane filter, and 20 µL of the resulting filtrate was injected for analysis. ZEN quantification was conducted by comparing the retention time and peak area of the samples against a ZEN standard curve, which demonstrated a robust linear correlation between peak area and concentration.

### 4.8. Biochemical Characterization

The optimum pH value for enzymatic activity was evaluated at 30 °C for 1 h, spanning a pH range from 2.0 to 9.0, employing various buffering systems. The buffers utilized were glycine-HCl (pH 2–3), sodium citrate (pH 3–4), sodium phosphate (pH 5–6), and Tris-HCl (pH 7–9). To assess pH stability, the isolated enzyme was preincubated at 30 °C for 1 h in buffers spanning a pH range of 2.0 to 9.0, devoid of substrate. The residual enzymatic activity towards ZEN was subsequently assessed under normal conditions of pH 8.0 and 30 °C for a duration of 1 h. The influence of temperature on the activity of pure lactone hydrolase was examined across a temperature range from 20 °C to 70 °C, at pH 8.0 for a duration of 1 h. The thermostability of Cr2zen was evaluated by quantifying the residual activity under standard test conditions (pH 8.0, 30 °C for 1 h) after preincubation of the enzyme at 30 °C, 40 °C, and 45 °C for different time durations without substrate. Enzyme kinetic experiments were performed with substrate doses varying from 0 to 20 mg/mL under optimal pH and temperature conditions. The kinetic parameters, *V_max_* and *K_m_*, were ascertained by applying non-linear regression analysis to fit the experimental data to the Michaelis-Menten equation. All experiments were conducted in triplicate to guarantee repeatability.

### 4.9. Statistical Analysis

Statistical analyses were performed on GraphPad Prism v10.6.1. Data are expressed as the mean ± standard error of the mean (SEM). Group differences were evaluated using two-way analysis of variance (ANOVA) for comparisons involving several groups. A *p*-value below 0.05 was deemed statistically significant.

## Data Availability

The original contributions presented in this study are included in the article. Further inquiries can be directed to the corresponding author.

## Supplementary Materials

The following supporting information can be downloaded at: https://www.mdpi.com/article/10.3390/toxins18010010/s1, Figure S1: PCR verification of the constructs; Figure S2: SDS-PAGE analysis of recombinant Cr2zen-100, Cr2zen-300, α-Cr2, Inv-Cr2, in *P. pastoris* after signal peptide processing; Figure S3: Codon optimization of *Ser-Cr2* to *Oser-Cr2*; Figure S4: Kex2 protease restriction sites of Oser-Cr2; Figure S5: PCR amplification confirmation of *Osercr2* and *Osercr2*-K196A constructs in *E. coli* and *Pichia pastoris*; Table S1: Specific primers used during the experiment; Table S2: Protein concentrations of recombinant Cr2-zen variants after expression; Table S3: HPLC quantification of zearalenone degradation by recombinant enzymes; Table S4: Analysis of protein concentration of codon optimized Oser-Cr2 strain.
