# Supplementary material for: Efficient Expression of Lactone Hydrolase Cr2zen for Scalable Zearalenone Degradation in Pichia pastoris
Source: Toxins (Basel). 2025 Dec 23;18(1):10. doi: 10.3390/toxins18010010 (PMC12846278; doi:10.3390/toxins18010010)
Supplement: Supplementary file 1 [file toxins-18-00010-s001.zip › toxins-4008439-supplementary.pdf]

Supplementary Materials

Efficient Expression of Lactone hydrolase Cr2zen for Scalable Zearalenone Degradation in *Pichia pastoris*

Mukhtar Ahmad[1#], Wang Hui[1#], Liu Xiaomeng[3#], Wang Shounan[3], Yin Tie[3], Deng Kun[1], Lu Caixia[1], Zhang Xiaolin[2*] and Jiang Wei[1*].

Mukhtar Ahmad. mukhtar.micro@gmail.com

Wang Hui. wh15666529202@163.com

Liu Xiaomeng. wsw_lxm2012@163.com

Wang Shounan. wangshounan@cofco.com

Yin Tie. yintie@cofco.com

Deng Kun. dengkun1103@163.com

Lu Caixia. bs20243020248@cau.edu.cn

Zhang Xiaolin. 77058@njnu.edu.cn

Jiang Wei. jiangwei01@cau.edu.cn

[1]State Key Laboratory of Animal Biotech Breeding, College of Biological Sciences, China Agricultural University, Beijing 100193, China.

[2]College of Food Science and Pharmaceutical Engineering, Nanjing Normal University, Nanjing 210023, Jiangsu Province, P.R. China;

[3]COFCO Nutrition and Health Research Institute Co., Ltd., Beijing 102209, P.R. China

[#] These authors contributed equally to this work

*Corresponding Author E-mail address. jiangwei01@cau.edu.cn

Contents:

Fig. S1. PCR verification of the constructs.

Fig. S2. SDS-PAGE analysis of recombinant Cr2zen-100, Cr2zen-300, α-Cr2, Inv-Cr2 in *P. pastoris* after signal peptide processing.

Fig. S3. Codon optimization of *Ser-Cr2* to *Oser-Cr2*.

Fig. S4. Kex2 protease restriction sites of Oser-Cr2.

Fig. S5. PCR amplification confirmation of *OserCr2* and *OserCr2-K196A* constructs in *E. coli* and *Pichia pastoris*.

Table. S1. Specific primers used during the experiment.

Table. S2. Protein concentrations of recombinant Cr2-zen variants after expression.

Table. S3. HPLC quantification of zearalenone degradation by recombinant enzymes.

Table. S4. Analysis of protein concentration of codon optimized Oser-Cr2 strain.

Supplementary Materials

Supplementary Table S1: Specific primers used during the experiment.

Primers Name	Base Sequence (5' to 3')
Na-F	AAAAAACAACTAATTATTCGAAACGGAATTCATGCGTACTAGATCTACT
Na-R	AGTAGATCTAGTACGCATGAATTCCGTTTCGAATAATTAGTTGTTTTTT
Ser-Cr2-F	TATTCGAAACGGAATTCATGGTCGCTTGGTGGTCTTTGTTTCTGTACGGTCTTCAGGTC
Ser-Cr2-R	AGTAGATCTAGTACGCATAGCAGGTGCAGCGACCTGAAGACCGTACAGAAACAAAGACC
Inv-Cr2-F	TTATTCGAAACGGAATTCATGCTTTTGCAAGCTTTCCTTTTCCTTTTGGCTGGTTTTGC
Inv-Cr2-R	AGTAGATCTAGTACGCATTGCAGATATTTTGGCTGCAAAACCAGCCAAAAGGAAAAGGA
5' AOX	GACTGGTTCCAATTGACAAGC
3' AOX	GCAAATGGCATTCTGACATCC

List of primer names and nucleotide sequences (5′–3′) used for cloning, colony PCR verification, and expression analysis of Cr2zen variants and vector-specific targets. Primer sequences are given exactly as synthesized.
Abbreviation: *AOX*, alcohol oxidase.

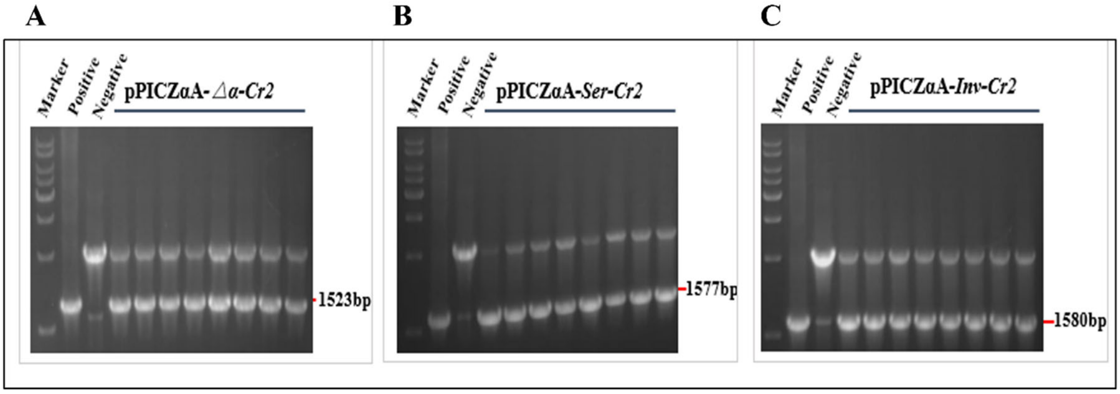

Supplementary Figure S1: PCR verification of the constructs.

Representative agarose-gel image showing PCR verification of cloned constructs. Lanes: marker (1 kb), positive control, negative control, and experimental samples. PCR was performed using the primers listed in Table S1. Bands correspond to expected amplicons for (A) *α-Cr2,* (B) *Ser-Cr2*, and (C) *Inv-Cr2*.

Supplementary Table S2. Protein concentrations of recombinant Cr2zen variants after expression.

Enzyme	Protein concentration (mg/mL)
Cr2-zen 100	0.014 ± 0.021
Cr2-zen 300	0.063 ± 0.009
Ser-Cr2	0.036 ± 0.011
Inv-Cr2	0.015 ± 0.001
△α-Cr2	0.086 ± 0.045

Protein concentrations measured for recombinant Cr2zen variants (Cr2-zen 100, Cr2-zen 300, Ser-Cr2, Inv-Cr2 and α-Cr2) following expression and purification. Values are reported as mean ± standard deviation from three independent biological replicates (n = 3). Concentrations were determined by Bradford assay using bovine serum albumin as standard.

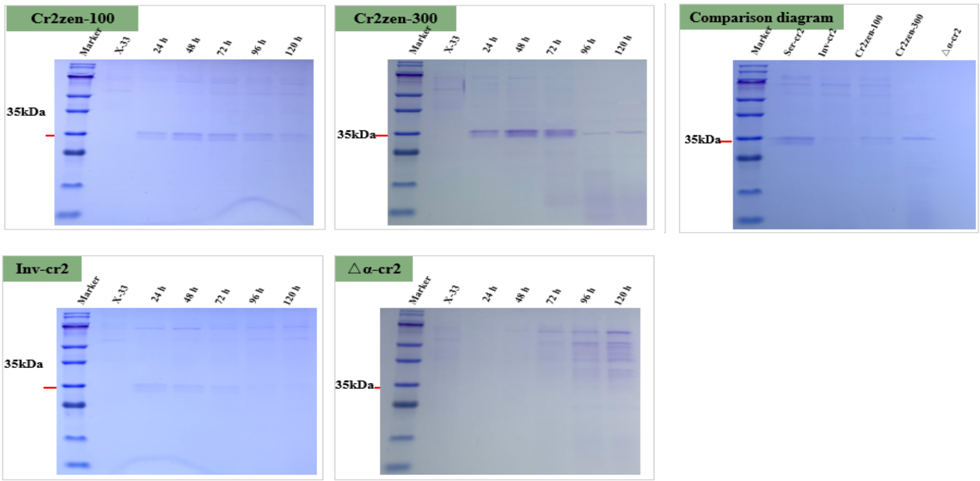

Supplementary Figure S2: SDS-PAGE analysis of recombinant Cr2zen-100, Cr2zen-300, α-Cr2, Inv-Cr2 after signal peptide processing.

SDS–PAGE gel showing expression and apparent molecular weights of Cr2zen-100, Cr2zen-300, α-Cr2 and Inv-Cr2 crude enzyme produced in *P. pastoris* after signal peptide cleavage, with comparison gel. Lanes: molecular weight marker (10–180 kDa); X-33 as ontrol, and crude lysates. Observed bands are indicated with estimated molecular weights (kDa). Gel is representative of three independent expression experiments. Samples were collected at 24-h intervals (0–120 h).

Supplementary Table S3: HPLC quantification of zearalenone degradation by recombinant enzymes.

Enzyme	ZEN Concentration (ng/mL)
Control	382.07 ± 0.71
Cr2zen-100	313.68 ± 5.19
Cr2zen-300	216.97 ± 0.80
Ser-Cr2	70.59 ± 2.74
Inv-Cr2	338.29 ± 5.11
△α-Cr2	346.46 ± 4.94

Functional activity of recombinant Cr2zen variants (Cr2zen-100, Cr2zen-300, Ser-Cr2, Inv-Cr2, and α-Cr2) and control (no enzyme) assessed by HPLC-based quantification of residual ZEN concentration. Data are presented as mean ± standard deviation (ng/mL) from three independent assays (n = 3).

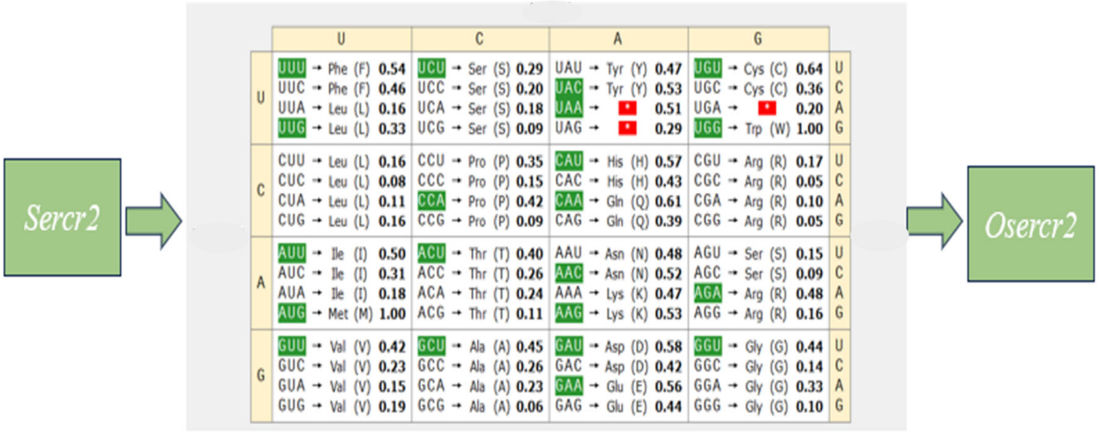

Supplementary Figure 3: Codon optimization of *Ser-Cr2* to *Oser-Cr2*.

Nucleotide alignment and summary of codon-usage metrics comparing the native Ser-Cr2 sequence to the codon-optimized Oser-Cr2 sequence. Codon changes were introduced to improve expression in the heterologous host; positions of non-synonymous changes are indicated.

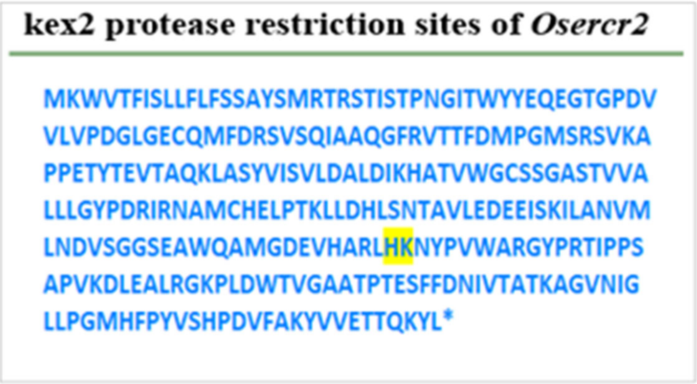

Supplementary Figure S4: Kex2 protease restriction sites of Oser-cr2.

Schematic and sequence view showing predicted Kex2 recognition cleavage sites in the codon-optimized Oser-Cr2 precursor.

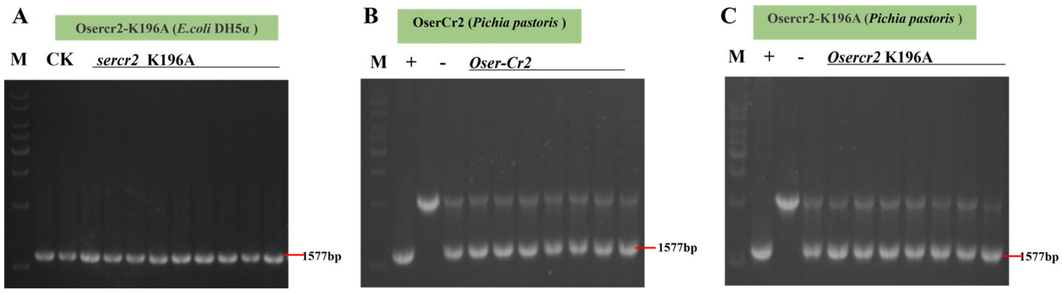

Supplementary Figure S5. PCR amplification confirmation of *Oser-Cr2* and *OserCr2-K196A* constructs in *E. coli* and *Pichia pastoris*.

(A) Colony PCR of *OserCr2-K196A* transformants in *E. coli* DH5α. (B) Colony PCR verification of *OserCr2* integration in *P. pastoris*. (C) Colony PCR verification of *OserCr2-K196A* integration in *P. pastoris*. Lane M: DNA ladder (1 kb); Lanes 1–8: independent transformant colonies; red arrow indicates the expected amplicon corresponding to the *OserCr2*, *OserCr2-K196A* gene fragment.

Supplementary Table S4: Analysis of protein concentration of codon optimized Oser-Cr2 strain

Enzyme	Protein Concentration (mg/mL)
Ser-Cr2	0.116 ± 0.001
Oser-Cr2	0.125 ± 0.004
Oser-Cr2-K196A	0.092 ± 0.014

Protein concentration of the codon-optimized Oser-Cr2 strain and its variants. Concentrations were determined after expression, and values are presented as mean ± standard deviation (mg/mL) from three independent biological replicates (n = 3). Concentrations were determined by BSA assay method.
